# Characteristics of TIA and its management in a tertiary care hospital in Pakistan

**DOI:** 10.1186/1756-0500-1-73

**Published:** 2008-08-29

**Authors:** Ayeesha Kamal, Farhad Khimani, Rushna Raza, Sahar Zafar, Salman Bandeali, Sayeedullah Jan

**Affiliations:** 1 Assistant Professor Neurology, Director Stroke Service, Aga Khan University Hospital, Karachi, Pakistan

## Abstract

**Background:**

Transient ischemic attack (TIA) is described as a brief episode of neurological dysfunction caused by focal brain ischemia, with clinical symptoms typically lasting less than an hour, and without evidence of acute infarction. Recent studies depict TIA as a particularly unstable condition. Risk of stroke is greater than 10% in the first 90 days after an index TIA. The presentation, prognosis and intervention for TIA have not been reported in South-Asians in a developing country.

**Method:**

A retrospective chart review was done for 158 patients who were admitted with the diagnosis of TIA, as defined by ICD 9 code 435, from January 2003 to December 2005 at the Aga Khan University Hospital, Karachi, Pakistan. The data was entered and analyzed in SPSS version 14.0.

**Findings:**

Among 158 patients, 57.6% were male and 41.1% were female. The common presenting symptoms were motor symptoms (51.3%), speech impairment (43%), sensory impairment (34.8%) and loss of balance/vertigo (29.1%). The median delay in presenting to the hospital was 4 hours. Those with motor symptoms were found to present earlier. The study showed that only 60.8% of all the patients presenting with TIA received any immediate treatment out of which 44.7% received aspirin. Neuroimaging was used in 91.1% of the patients. Of all the TIA patients 9.1% converted to stroke with 50% doing so within the first 24 hours.

**Conclusion:**

The natural history of TIA from this developing nation is comparable to international descriptions. A large percentage of patients are still not receiving any immediate treatment as recommended in available guidelines, even in a tertiary care hospital.

## Background

Transient Ischemic attacks (TIA) represent an opportunity to prevent strokes. Although the risk factors, evaluation and secondary prevention of TIA and stroke are comparable, TIA is conventionally considered a less urgent manifestation and therefore receives leisurely intervention [1,2]. Whether an intervention can prevent the development of stroke in patients presenting with TIA, has been an area of significant debate and research. In the interim, given the natural history, proper care and management should be provided to all patients with TIA [3,4]. The EXPRESS study has shown that early initiation of existing treatments after TIA or minor stroke was associated with an 80% reduction in the risk of early recurrent stroke [5].

TIA has been described as a stroke that lasts less than 24 hours. The American TIA Working Group proposed a new definition which states that a transient ischemic attack is a brief episode of neurologic dysfunction, caused by local brain or retinal ischemia, with clinical symptoms typically lasting less than one hour, and without evidence of cerebral infarction[6]. The early estimated risk of stroke after a TIA is 8–12% at seven days and 11–15% at one month [6,7]. A study from California done on the short term prognosis of transient ischemic attacks revealed that in a 90 day period after a TIA, one in nine patients had a stroke and half of all the strokes occurred in the first 2 days [8].

Stroke is the leading cause of sustained neurological disability in the world [9]. Two thirds of all strokes now occur in the developing world. South Asia represents a quarter of the emerging world and harbors 20% of the global stroke population [9]. Approximately 15% of ischemic strokes are preceded by a TIA [7]. Thus TIA represents an intervention opportunity in those most at risk.

In Pakistan, non communicable disease now accounts for 41% of total disease burden. The National Health Survey of Pakistan (NHSP) estimates that Hypertension – the single most preventable cause of stroke and TIA – affects one in three adults aged greater than 45, and 19% of the population aged 15 and above. The NHSP showed that DM is present in 35% of adults greater than 45 years of age [10]. Coronary artery disease can cause cardio embolic stroke and is a surrogate for atherosclerosis in the cerebrovascular system: a population based cross sectional survey showed a prevalence of 1 in 4 middle aged adults with men and women at equal risk [11]. The overall prevalence of obesity is 28% in women and 22% in men. The prevalence of tobacco use is 40% in men and 12% in women [12]. Various other factors, like unhealthy dietary patterns, low socio-economic strata and genetic predisposition in certain population groups, have repeatedly been identified [9,13-16]. Adding to this, other challenges like the lack of knowledge of TIA symptomatology, diagnosis and awareness of its serious implication at the primary health care level, and where these patients may present to, make the awareness and proper management of TIA patients all the more important [17,18]. The presentation, natural history and prognosis of TIAs are not known for Pakistan, where the risk profile and population is very different from Caucasian cohorts.

The objective of this study is to describe the base line characteristics, presentation and the current management of TIA events in daily clinical practice in a tertiary care hospital in a developing country. This information would help prevent stroke in those most at risk of developing this disease and will provide a comparison with findings from the Western countries.

## Methods

### Study Design

A retrospective chart review was conducted for inpatients who were admitted and diagnosed with TIA between January 2003 and December 2005 at the Aga Khan University (AKU), a teaching hospital in Karachi, Pakistan. Patients who were admitted and diagnosed with TIA, as defined by ICD 9 code, were included in this study. The definition of TIA was the sudden onset of neurologic deficit respecting a vascular territory with resolution of symptoms within 24 hours. The majority of TIAs actually resolve within 60 minutes, and most of these resolve within 30 minutes. Levy showed that the likelihood that symptoms will resolve completely within 24 hours is less than 15 percent if symptoms last more than 1 hour [19]. In the emergency room, all patients who present with TIA are offered admission for 24 hours.

These patients were diagnosed with TIA by the neurologist on service. The patients were evaluated in the emergency room and admitted in the hospital via the emergency room, or picked up as consultations from other in patient services e.g. TIA on internal medicine service. The neurologic consultant assigned the vascular diagnosis and the hospital coding service assigned the ICD 9 code number. 200 charts were selected after the approval of the Ethical Review Committee (AKU-ERC). 35 charts were either irretrievable or had incomplete data and hence, were excluded. Out of the remaining 165, seven were TIA mimics and not true cases of TIA, therefore these were excluded.

### Study Setting

This is a hospital based survey conducted at the Aga Khan University Hospital (AKUH). AKUH is a tertiary care hospital in Karachi, Pakistan, with a total patient population of 334,393 outpatients and 32,500 inpatients yearly. Karachi is an urban city with a population of 20 million. This study population reflects the reported TIA rates of a non rural, multiethnic South Asian population in transition. During the study period (January 2003– December 2005) there were 1036 admissions for stroke. The stroke and TIA admissions came from all over the city, and from households that would be described as lower to middle class urban city dwellers. Thus this study is generalisable only as far as Asian "population in transition" (Omran's Theory of rural population being urbanized) [20], which may be most at risk for non communicable disease. In the rural areas there are government run district health centers and the presentation and total numbers of TIAs are not known for Pakistan. There is also a lack of community based statistics.

### Data collection tool

The main elements of the questionnaire, designed after a thorough review of literature, were the presenting symptoms, co-morbid illnesses, history of use of anticoagulants and antiplatelet agents, laboratory tests carried out, emergency room work up and the initial treatment, management in the stroke units and further follow-up of the patients to a minimum of three months with new stroke and its workup details. Risk factor definitions were

a) Hypertension defined either as:

1. Blood pressure >140/90 mmHg or >130/80 mmHg for diabetic patients (before stroke or at least one week after stroke), or

2. Known hypertension and being treated with antihypertensives [21].

b) Overweight: BMI 25.0 – 29.9 kg/m^2 ^as defined by WHO [22]

Obese: BMI ≥30.0 kg/m^2 ^as defined by WHO [22].

c) Diabetes Mellitus as defined by any one of the following:

1. Fasting plasma glucose level at or above 126 mg/dL (7.0 mmol/l)

2. Plasma glucose at or above 200 mg/dL (11.1 mmol/l) 2 hours after a 75 gram oral glucose load as in a GTT.

3. Random plasma glucose at or above 200 mg/dL (11.1 mmol/l)

4. Known diabetic being treated with dietary modification, medication or both.

d) Smoker: >1 cigarette/day

Ex-smoker: Stopped smoking ≥2 years ago

e) Dyslipidemia defined as:

1. Known case of dyslipidemia on treatment or TG > 240 mg/dL

2. Hypercholesterolemia: LDL > 100 mg/dl [23].

### Data collection, analyses and follow up

The data was collected by the sub-investigators and then entered and analyzed in SPSS 14.0. A qualified stroke neurologist classified the mechanism responsible for TIA according to the TOAST classification [24].

Descriptive statistics were run for age, delay in presentation, and duration of hospital stay, radiological data and laboratory tests. Cross-tabs were carried out and Chi-square tests were performed to find any association of delay in presentation, symptom at presentation, age and gender with management received, and for the comorbid illnesses, risk factors and initial treatment received by the TIA patients in the emergency room with recurrent TIA or new stroke development.

All patients were given a 90 day follow up appointment in the outpatient clinic and telephone contact was established with the patients so as to remind them of their follow up appointments. Since all charts in the hospital for a single patient are linked (there are no separate inpatient and outpatient charts) it is possible to review follow up data as well.

## Results

Among 158 patients, 57.6% were male and 41.1% were female. The mean age of the patients was 60.23 ± 13.14 but the median age was 60, and the mode of the data for age was 58. The most common presenting symptom was motor impairment followed by speech difficulty. Table 1 depicts the presenting symptoms of the patient population. The median for the delay in presentation to the hospital was 4 hours and the mean was 20 hours and 46 minutes.

**Table 1:** Presenting symptoms of the patient cohort

**Presenting symptoms**	**Frequency(%)**
Motor symptoms	51.3
Sensory symptoms	34.8
Difficulty or loss of speech	43.0
Vertigo or loss of balance	29.1

Table 2 depicts the comorbid illnesses and risk factors of the patient population as well as a comparison with the sub group that developed new stroke.

**Table 2:** A comparison of risk factors in TIA patients versus new stroke patients

**Characteristics**	**Frequency(%)** **TIA patients**	**Frequency(%)** **New stroke patients**
Hypertension	122(77.2)	12(80.0)
Diabetes	61(38.6)	4(26.7)
Stable angina	10(6.3)	2(13.3)
Unstable angina	9(5.7)	2(13.3)
Previous myocardial infarction	23(14.6)	0(0.0)
Congestive heart failure	5(3.2)	0(0.0)
Peripheral vascular disease	2(1.3)	5(33.3)
Hypercholesterolemia	72(45.6)	6(40.0)
Obesity	18(11.4)	1(6.7)
Coagulopathy	6(3.8)	1(6.7)
Atrial fibrillation	8(5.1)	1(6.7)
History of smoking	33(20.9)	3(20.0)
Prior use of at least one Medication (for any disease)	119(75.3)	13(86.7)

Table 3 depicts the immediate management and interventions received by the patients.

**Table 3:** Immediate therapeutic and diagnostic interventions performed on the patient population

**Initial management**	**Frequency(%)**
Percentage receiving any form of initial treatment	60.8
No immediate treatment	39.2
Initial aspirin	27.2
Other antiplatelet (Clopidogrel and Persantin)	12.7
Statins	1.9
Heparin	6.3
Low molecular weight heparin	8.2

**Radiological diagnostic modalities**	**Frequency(%)**

One or more radiological diagnostic modalities	91.1
MRI	53.2
Carotid dopplers	47.5
MRA	41.8
CT Scan	27.2
Echocardiography	57.6

**Initial counseling**	**Frequency(%)**

Documented counseling for smoking cessation	8.2

The most common type of TIA came out to be of undetermined and under investigated type (24.1%), followed sequentially by presumable cardioembolic (20.3%), large artery atherosclerosis (18.4%), probable lacunar warning syndrome (15.8%) and truly undetermined (1.3%).

Out of the total 158 patients presenting with TIA, 9.5% developed stroke in the first year. Among them, 50% developed the stroke within 24 hours and 83.4% within 90 days following their initial TIA. All the strokes were diagnosed in hospital. These were all ischemic strokes. Although we did not look at ICH or SAH as predefined outcomes, these were not reported in the small number of outcome events that were studied.

Significant associations were found between male gender and high LDL (p = 0.001), high triglycerides (p = 0.001) and positive history of smoking (p = 0.00). Increasing age was found to be associated with more sensory symptoms at presentation (p = 0.04). We found a significant association between the development of a new stroke after the initial TIA event with positive history of unstable angina (p = 0.00), high LDL, elevated triglycerides, low HDL (p = 0.01), time delay to presentation after onset of symptoms (p = 0.003) and with the length of hospital stay (p = 0.042). There was a positive association between speech symptoms at presentation and initial aspirin administration (p = 0.039). We found a negative association between initial treatment with clopidogrel and heparin, with new stroke (p = 0.021).

We did not find any significant association between new stroke occurrence with administration of aspirin (p = 0.2) or statin (p = 1.0) as an initial treatment in the emergency room on presentation with TIA. This may be due to the small number of outcome events.

## Discussion

The high risk of stroke after a transient ischemic attack supports an approach involving rapid evaluation and initiation of treatment. Consensus guidelines on the management of TIA have been promulgated by the American Heart Association AHA and the National Stroke Association [3]. With regard to finding the cause and diagnosis of TIA, our center has principally followed the AHA and NSA guidelines. We found that about 91% of the patients were diagnostically evaluated via at least one radiographic imaging modality. These diagnostic tests were done to identify or exclude etiologies of TIA requiring specific therapy, to assess modifiable risk factors, and to determine prognosis. Head MRI and CT were done in 53.2% and 27.2% of the cases respectively. The guidelines also recommend imaging the carotid arteries in all cases but in our study only 47.5% of patients underwent carotid imaging. The reason for the low number of carotid dopplers may be the perception held by the doctors that our population is different in terms of stroke causation. This perception is based on studies that suggest that Asians have more intracranial atherosclerosis [25,26]. The figures for neuroimaging are comparable with Western figures, as a national study on emergency department visits for TIA in the United States revealed that CT scan was performed on 56% patients and MRI on less than 5% of patients, though over the period of the study there was an increase in the trend to perform neuroimaging [27]. Another study conducted in 4 regional stroke centers in Ontario revealed that diagnostic interventions were underutilized. CT scanning was performed in 58%, carotid Doppler ultrasonography in 44%, and MRI in 3% of patients [28].

Transient Ischemic attack, regarded as being the warning sign for stroke needs prompt evaluation and management [6]. Majority of the patients in our study were provided robust evaluation but fewer received any immediate treatment, among which aspirin was the most common medicine administered. About 62.8% received immediate treatment, of which 44% got aspirin(27.2% of the total) while 19% got other anti-platelet medications. Statins and heparin constituted only a meager 12% together. The use of aspirin as an initial treatment is in accordance with the AHA guidelines. Aspirin use in the early phase after stroke or TIA reduces the risk of recurrence [29]. However, we did not find any significant association between aspirin and the risk of stroke in our study. This may be because our sample size was very small with only 15 patients developing new stroke after the initial TIA. 39.2% of our patients received no immediate medical treatment which compares with the national study on emergency department TIA management in the United States in which 42% of patients received no immediate medical treatment [27].

It has been reported in several studies that age > 60 yrs is a risk factor for stroke after TIA. The ABCD and California risk score systems have taken age > 60 yr as a significant risk factor to predict future stroke after TIA [30]. Our study did not find any association between age and stroke occurrence. Although the mean age of our patients was 60.23 ± 13.14, but the median age was 60 and the mode of the data for age was 58. This shows that in our region much younger population suffers from TIA and stroke. We attribute it to the dietary and genetic factors as previously described. This aspect should be further explored in terms of the factors behind this difference in the age at presentation.

The most common risk factors prevalent in descending order of frequency were hypertension, hypercholesterolemia and diabetes respectively. This emphasizes the need to address these co-morbid illnesses for secondary prevention of stroke. Smoking has been found to be associated with stroke. In our study 20.9% were smokers but the documented counseling for smoking cessation was done in only 8.2% of the patients. This raises an important issue regarding prevention of stroke. We propose that all the smokers should be counseled for cessation in-hospital and on outpatient follow-up visits. Such an approach might help reduce the incidence of stroke among TIA patients that is attributable to smoking.

Raised LDL and triglycerides were significantly associated with male gender (p = 0.001). In our study more male patients presented with TIA which is contrary to previous studies which identify female gender as a risk factor. The outcome of patients with TIA depends on a number of factors like the initial treatment received and comorbid illnesses. The delay in accessing hospital care and receiving treatment is also important. Our study showed that increased time delay of symptoms was associated with a prolonged hospital stay (p = 0.042) and increased incidence of stroke (p = 0.003).

We were unable to deduce significant associations with risk factors and the occurrence of new stroke, probably because our sample size was small. We therefore recommend that further studies be carried out to find out various risk factors for stroke.

In our study, 9.5% of TIA patients developed stroke, which is comparable to the international figure of 10.5% stroke conversion. Our study, also verified the findings in previous studies that the greatest risk of stroke after TIA is in the first 48 hours. 9.5% of our patients developed stroke and about 50% did so in the first 24 hours. There is significant underutilization of investigative modalities and the immediate medical management options for TIA. This adds to the preexisting lack of attention to preventive medicine. Data from Pakistan from the WHO_ PREMIS study that looked at patients that accessed primary care facilities – showed under utilization of prevention practices of quitting smoking, exercise, reduction of dietary salt intake and limited use of aspirin (83%), B Blockers (35%), ACEI (43%) and Statins (2.3%) [31]. This study was limited to patients who visit outpatients facilities and are likely to differ from the general population in their health seeking behavior – the national situation regarding prevention is likely to be far worse than this sample.

The stroke epidemic of the developing world disables individuals in their prime of life, and is mostly preventable [32]. TIA represents an opportunity to intervene in those most at risk. 50% of the stroke conversions in our study occurred within the first 24 hours. A large percentage of these patients did not receive any immediate treatment. Hence a rapid evaluation is immensely important to prevent stroke and permanent neurologic sequelae in these patients and to thwart the expanding stroke morbidity and mortality in the developing world.

## Authors' contributions

AK conceived, designed the study and monitored its data quality, wrote manuscript and was involved in all stages of the study. FK, RR, SB, SJ collected data, drafted the manuscript and performed analysis. SZ assisted in formulating the final manuscript and performed exploratory analysis after review. All authors read and approved the final manuscript.
